# Poly-arginine-18 peptides do not exacerbate bleeding, or improve functional outcomes following collagenase-induced intracerebral hemorrhage in the rat

**DOI:** 10.1371/journal.pone.0224870

**Published:** 2019-11-07

**Authors:** Lane Liddle, Ryan Reinders, Samantha South, David Blacker, Neville Knuckey, Frederick Colbourne, Bruno Meloni

**Affiliations:** 1 Department of Psychology, University of Alberta, Edmonton, Alberta, Canada; 2 Office of Research Enterprise, The University of Western Australia, Western Australia, Australia; 3 Perron Institute for Neurological and Translational Science, Nedlands, Western Australia, Australia; 4 Centre for Neuromuscular and Neurological Disorders, University of Western Australia, Nedlands, Western Australia, Australia; 5 Department of Neurology, Sir Charles Gairdner Hospital, Nedlands, Western Australia, Australia; 6 Department of Neurosurgery, Sir Charles Gairdner Hospital, Nedlands, Western Australia, Australia; 7 Neuroscience and Mental Health Institute, University of Alberta, Edmonton, Alberta, Canada; University of Warwick, UNITED KINGDOM

## Abstract

**Background:**

Cationic arginine-rich peptides (CARPs) have demonstrated neuroprotective and/or behavioural efficacy in ischemic and hemorrhagic stroke and traumatic brain injury models. Therefore, in this study we investigated the safety and neuroprotective efficacy of the CARPs poly-arginine-18 (R18; 18-mer of arginine) and its D-enantiomer R18D given in the acute bleeding phase in an intracerebral hemorrhage (ICH) model.

**Methods:**

One hundred and fifty-eight male Sprague-Dawley rats received collagenase-induced ICH. Study 1 examined various doses of R18D (30, 100, 300, or 1000 nmol/kg) or R18 (100, 300, 1000 nmol/kg) administered intravenously 30 minutes post-collagenase injection on hemorrhage volume 24 hours after ICH. Study 2 examined R18D (single intravenous dose) or R18 (single intravenous dose, plus 6 daily intraperitoneal doses) at 300 or 1000 nmol/kg commencing 30 minutes post-collagenase injection on behavioural outcomes (Montoya staircase test, and horizontal ladder test) in the chronic post-ICH period. A histological assessment of tissue loss was assessed using a Nissl stain at 28 days after ICH.

**Results:**

When administered during ongoing bleeding, neither R18 or R18D exacerbated hematoma volume or worsened functional deficits. Lesion volume assessment at 28 days post-ICH was not reduced by the peptides; however, animals treated with the lower R18D 300 nmol/kg dose, but not with the higher 1000 nmol/kg dose, demonstrated a statistically increased lesion size compared to saline treated animals.

**Conclusion:**

Overall, both R18 and R18D appeared to be safe when administered during a period of ongoing bleeding following ICH. Neither peptide appears to have any statistically significant effect in reducing lesion volume or improving functional recovery after ICH. Additional studies are required to further assess dose efficacy and safety in pre-clinical ICH studies.

## Introduction

Intracerebral hemorrhage (ICH) is a devastating stroke characterized by cerebral vascular rupture, and bleeding within the parenchyma. ICH accounts for about 10–15% of all strokes [1]. Despite advances in our understanding of the disease, the 30-day mortality of ICH has remained unchanged at about 40%; those that survive are often left with persistent functional impairment [2]. The high disability rates following ICH are even more concerning given the fact that there are no clinically available neuroprotective therapies for ICH.

Approaches to neuroprotection tend to focus on reducing either primary and/or secondary injury following ICH [3]. Primary injury occurs as blood dissects through the parenchyma, causing mechanical destruction and displacement of brain structures. One major approach to reduce primary injury is to limit hemorrhage volume (e.g., by blood pressure reduction [4] or augmentation of hemostasis [5]). These therapies are based on the fact that hematoma volume is a robust determinant of death and disability in ICH patients. Very large hematomas are associated with raised intracranial pressure and brain herniation which affects vital autonomic structures [6]. Approaches to limit secondary injury typically focus on targeting the injurious sequelae triggered by the presence of blood and its breakdown products in the parenchyma (e.g., dampening the neuroinflammatory response) [7]. Some of these mechanisms of injury overlap with traumatic and ischemic brain insults, leading many to take therapies from those fields to test in ICH models (e.g., anti-inflammatory drugs, free radical scavengers).

Recently, our laboratory has demonstrated that a class of peptide known as cationic arginine-rich peptides (CARPs) have potent neuroprotective properties, with increasing effectiveness as peptide arginine content increases, peaking at 15 to 18 residues for poly-arginine peptides [8]. In particular, poly-arginine-18 (R18; 18-mer of arginine; net charge +18) is strongly neuroprotective in *in vitro* neuronal excitotoxicity and ischemic stroke models and more recently in models of traumatic brain injury [9–12]. The quality of the evidence for use of CARPs in acute ischemic stroke is relatively strong. For example, even when administered at a clinically-relevant delay, CARPs have demonstrated neuroprotective efficacy in multiple rodent models, and in higher-order nonhuman primates [9–11].

Evidence suggests that CARPs may confer neuroprotection via at least three pathways. First, CARPs reduce excitotoxic injury by inhibiting calcium influx following cellular injury [13,14]. Second, CARPs may prevent the activation of apoptotic pathways by preserving mitochondrial function [15], and protecting mitochondrial architecture [16]. Finally, CARPs inhibit proteolytic enzymes that activate matrix metalloproteinases (MMPs) [17,18]. MMP activation is associated with blood-brain barrier disruption, edema, hemorrhage, and leukocyte activation and infiltration [19]. Hence, CARPs may be a potential treatment for secondary injury following ICH because excitotoxicity [20–22], mitochondrial dysfunction [23], MMP activation [24,25], and apoptosis [26] contribute to ICH-related brain injury. Indeed, several studies have demonstrated that different CARPs do not exacerbate bleeding, but they did reduce cerebral edema and inflammation, and improve functional outcomes in rodent models of ICH (Table 1). None of those studies examined long-term histological outcomes following CARP administration (i.e., lesion volume assessment following a > 1-week post-ICH survival).

**Table 1:** Studies using CARPs in ICH models.

Peptide name, sequence & net charge	Proposed target	Injury model	Route & treatment schedule	Dose	Outcomes after ICH	Reference
JNKI-1-TATD tdqsrpvqpflnlttprkpr-pp-rrrqrrkkrg-NH_2_; +12	JNK	Mouse: collagenase.	1) IV: 3 h post-ICH	1) 25 nmol/kg	• Improved neurological score on d 1, but not d 2 or 5. Reduced lesion volume on d 2, and hemisphere swelling on d 2 & 5.	[57]
COG1410 Ac-ASAibLRKLAibKRLL-NH_2_; +4	Unknown	Mouse: collagenase.	1) IV: 0.5 h post-ICH 2) IV: 0.5 h post-ICH 3) IV: 0.5, 1, 2, or 4 h post-ICH 4) IV: 0.5 h post-ICH & daily for 5 d	1)355, 710, 1420, 2840 nmol/kg 2) 1420 nmol/kg 3) 1420 nmol/kg 4) 1420 nmol/kg	• All doses improved rotarod performance at 5 d. • Reduced cerebral edema & inflammatory proteins, but not hematoma volume at 24 h. • 0.5, 1 & 2 h treatment improved rotarod running at 5 d. • Reduced microglia activation at 5 d.	[58]
		Mouse (male & female; Apoe3 & Apoe4): collagenase.	1) IV: 1 h post-ICH & daily for 7 d	1)1420 nmol/kg	• No reduction in hematoma volume at 24 h. Beneficial effects on rotarod (d 7) & Morris water maze (d 32) performance. Female Apoe3 mice benefited most.	[59]
CN-105 Ac-VSRRR-NH_2_; +3	Unknown	Mouse: collagenase.	1) IV: 2, 6, & 24 h post-ICH 2) IV: 4, 8, & 24 h post-ICH	1) 70 nmol/kg 2) 70 nmol/kg	• Reduced cerebral edema, but not hematoma volume at 24 h. • May improve neuroseverity score & rotarod performance on d 5, & Morris water maze on d 32. Reduced microglia activation, CA3 neuron loss at 5 d.	[60]
Apelin 13 QRPRLSHKGPMPF; +3.1	Apelin receptor	Mouse: collagenase.	1) ICV: immediately post-ICH	1) 50 μg/5 μL (32 nmoles or 1070 nmol/kg)	• Improved motor outcomes on d 1–6 & reduced cerebral edema at 12, 24 & 48 h. Reduced AQP4, MMP-9, caspase 3, & maintained Bcl-2 levels at 24 & 48 h. Reduced TUNEL positive cells at 48 h.	[61]
TAT-NR2B9c YGRKKRRQRRR-KLSSIESDV; +7	PSD95/ nNOS	Rat: collagenase. Rat: autologous blood	1) IV: 0.5 h post-ICH 2) IV: 0.5 h post-ICH & once every 3 d for 22–26 d 3) IV: 0.5 h post-ICH	1) 1032 nmol/kg 2) 1032 nmol/kg 3) 1032 nmol/kg	• Reduced cerebral cell death, edema, IL-1, IL-17, caspase, MMP-9 activation, but not hematoma volume at 24 h. Improved neurobehavioural scores at 3 d. • Improved water maze performance at 22–26 d. • Improved neurobehavioural scores at 3 d.	[62]

1. JNKI-1-TATD also known as XG-102, CN-105 derived from COG1410 and TAT-NR2B9c also known as NA-1. Lower case = D-amino acid.

2. JNK = c-Jun N-terminal kinase.

3. Collagenase injection into striatum.

h = hours, d = day.

Therefore, given the previous studies demonstrating the benefits of other CARPs in ICH, the accumulating evidence for neuroprotective efficacy of R18 in ischemic stroke, and the potential for early field administration of neuroprotective agents by ambulance paramedics before stroke subtype confirmation, we sought to evaluate whether R18 and its D-enantiomer R18D were safe to administer following collagenase-induced ICH in rats, and whether it showed signs of neuroprotective or behavioural efficacy. In Study 1, we hypothesized that R18 and R18D would not impact hematoma volume in the acute phase. In Study 2, we hypothesized that R18 and R18D would reduce brain injury and improve functional outcomes when assessed long-term after ICH. Long-term functional and histological assessments are important translational endpoints because they capture net injury and repair processes that evolve over days and weeks following ICH, and reduce the risk of drawing premature conclusions based upon observations obtained from short-term study endpoints [27,28]. This point is important, as progressive brain damage can be observed in the collagenase model using serial MRI for several weeks [27]. In all experimental studies, we used doses that have demonstrated neuroprotection in previous research in models of middle cerebral artery occlusion and hypoxic-ischemic encephalopathy [29,30].

## Methods

### Peptides used in the study

The R18 (H-RRRRRRRRRRRRRRRRRR-OH) and R18D (H-rrrrrrrrrrrrrrrrrr-OH) peptides were synthesized containing the L-isoform or the D-isoform amino acids, respectively (D-arginine represented by lower case). Peptides were synthesized by Mimotopes (Australia), purified by high performance liquid chromatography to at least 98% purity, and subjected to peptide hydrolysis and amino acid liquid chromatography analysis to obtain a precise measure of peptide content (Mimotopes). Two different batches of R18D (batch 1 and batch 2) were used for the hematoma volume assessment. Peptides were prepared in 0.9% sodium chloride for injection in a 650 μL volume in 1 mL syringes. Reconstituted peptides were stored at -20°C until use.

### Experimental animals

All studies were conducted at the University of Alberta, in accordance with the Canadian Council on Animal Care Guidelines. All procedures were approved by the Biosciences Animal Care and Use Committee at the University of Alberta. Surgical procedures were performed using isoflurane anesthesia with perioperative and intraoperative pain management. Animal health assessments were conducted at least twice daily.

**Fig 1:**
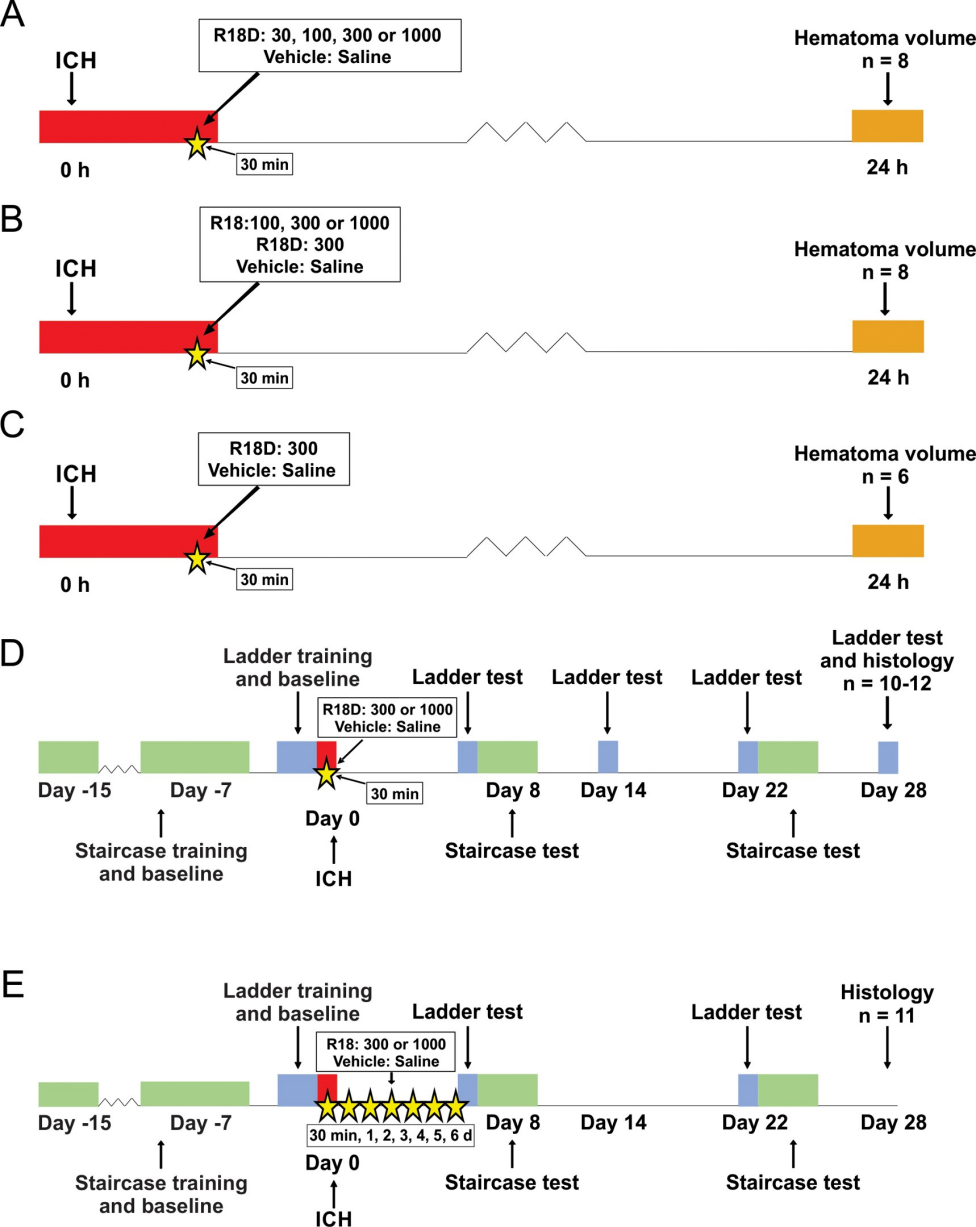
Experimental timeline (days relative to surgery) for safety and efficacy assessment studies. **A.** Study 1A: R18D dose-response safety study (n = 8 per group). **B.** Study 1B: R18 dose-response safety study (n = 8 per group). **C.** Study 1C: 300 nmol/kg R18D vs. saline safety study (n = 6 per group). **D.** Study 2A: 300 and 1000 nmol/kg R18D efficacy study (saline group, n = 10; 300 nmol/kg group, n = 12; 1000 nmol/kg group, n = 11). **E.** Study 2B: 300 and 1000 nmol/kg R18 efficacy study (n = 11 per group). Red bars represent the ICH induction period. Orange bars represent the hematoma assessment. Green bars represent the skilled reaching (Montoya staircase) task, and blue bars represent the horizontal ladder task. Yellow stars represent treatment time points for R18D, R18 or saline vehicle. In Study 1 (A,B,C): R18D, R18, and saline were administered into the femoral vein once, 30 minutes post-collagenase infusion. In Study 2A: R18D, and saline were administered once into the femoral vein 30-minutes post-collagenase infusion. In Study 2B: R18, and saline were administered into the femoral vein 30-minutes post-collagenase infusion, and additional doses were administered once intraperitoneally every 24 hours for a total of 7 doses.

One hundred and fifty-eight male Sprague Dawley rats (age: ~2 months; weight: 270–330 g) were obtained from Charles River (Saint Constant, QC). Ninety-two rats were used in Study 1 (hematoma volume assessment) and 66 rats were used in Study 2 (histology and functional assessment). Experimental details are provided in Fig 1.

Rats were group housed four per cage in a temperature and light controlled room with 12-hour light-dark cycles. Food (Purina Rodent Chow) and water were provided *ad libitum* except during the period of 90% free-feeding weight food deprivation for skilled-reaching training and testing. Rats were handled for 20 minutes (two 10-minute sessions over two days) prior to Study 2 to reduce stress.

### Study 1: Hematoma volume assessment

This study consisted of a dose response study for R18D (Study 1A) and R18 (Study 1B), with a confirmatory study using the 300 nmol/kg dose of R18D versus saline (Study 1C). R18D at the 300 nmol/kg dose was included in Study 1B and in Study 1C to determine any difference between rats receiving different batches of the peptide at this dose and saline. Before Study 1, we conducted a power-analysis to calculate our ability to detect a 10 μL increase in hematoma volume with 80% power, which would require 8 rats per group.

#### Study 1A

Animals received either a 30, 100, 300, or 1000 nmol/kg dose of R18D (batch 1), or saline vehicle 30-minutes after collagenase injection (n = 8 per group).

#### Study 1B

Animals received a 100, 300, or 1000 nmol/kg dose of R18, a 300 nmol/kg dose of R18D (batch 2) or saline 30-minutes after collagenase injection (n = 8 per group).

#### Study 1C

Animals received either a 300 nmol/kg dose of R18D (batch 1) or saline 30-minutes after collagenase injection (n = 6 per group).

### Study 2: Lesion volume and behavioural assessment

This study consisted of a dose response study for R18D (Study 2A) given once post-ICH, and dose response study for R18 (Study 2B) administered at multiple times after ICH.

#### Study 2A

Animals received either 300, 1000 nmol/kg of R18D (batch 1) or saline 30-minutes after collagenase injection (300 nmol/kg, n = 12; 1000 nmol/kg, n = 11; saline, n = 10).

#### Study 2B

Animals received either 300, 1000 nmol/kg of R18 or saline 30-minutes after collagenase injection, followed by daily injections for 6-days (n = 11 per group).

### Intracerebral hemorrhage, treatment administration and experimental groups

Intracerebral hemorrhage was induced via stereotactic infusion of one microliter of collagenase (0.6 U/μL; Type IV; Sigma, Oakville, Ontario, Canada) into the striatum using a protocol described in our previous publications [31]. In Study 1, all animals received ICH in the right striatum. In Study 2, animals received ICH in the striatum contralateral to their dominant forelimb. Forelimb dominance was assessed on the final 3 days of skilled reaching assessment. Isoflurane anesthesia was maintained following surgery to allow for femoral vein cannulation and subsequent infusion of R18, R18D, or saline vehicle (600 μL over 6 minutes). Following femoral infusion, the wound was sutured, the animal was placed in an individual recovery cage for monitoring and was returned to group housing after one hour of monitoring.

Animals were randomized to treatment groups at the time of surgery to minimize bias in training and baseline assessment. Randomization was achieved using a free, online tool (https://www.randomlists.com/team-generator). Experimenters were blinded to treatment group during all surgical procedures, data collection, and data analysis.

### Spectrophotometric hemoglobin assay

The impact of R18D or R18 on hematoma volume was assessed using a spectrophotometric hemoglobin assay developed by Choudhri and colleagues [32], with modifications as described in our previous publications [33]. Briefly, brains were extracted 24 hours following ICH induction and blood volume in the ipsilesional hemisphere and contralesional hemisphere was determined. Hematoma volume was computed by correcting for individual differences in cerebral vasculature as: *Hematoma volume = ipsilesional hemisphere blood volume−contralesional hemisphere blood volume*.

### Skilled reaching task

Animals were trained to reach for fortified banana pellets (45 mg; Bioserv, Flemington, NJ) in the Montoya staircase test [34]. Three days prior to training, animals were food deprived to 90% of their free feeding weight (assuming natural gains with age) in order to encourage reaching for pellets. During food deprivation, rats receive nutrition during training (from the fortified pellets) and during feeding following the last training session of each day (from rodent chow). Training and testing trials lasted 15 minutes and occurred twice daily (4 hours between sessions). Training was conducted for 15 days prior to ICH surgery. During each trial, animals had the opportunity to reach for a maximum of 21 pellets per side. The number of pellets consumed was calculated for each forelimb according to the following formula: *Pellets consumed* = 21−*pellets dropped−pellets remaining*. Baseline testing and forelimb dominance assessment occurred on the final 3 days of training. Food deprivation ended 4 days prior to ICH to ensure rats returned to free-feeding weight. Post-ICH testing occurred on days 8, 9, 10 and 22, 23, 24 with food deprivation commencing on days 7 and 21. The schedule for food deprivation, skilled-reaching training and testing were the same for Experiment 2A and 2B (Fig 1). The Montoya staircase was our primary behavioural endpoint because we have previously shown that the Montoya staircase task is relatively sensitive to the relationship between lesion volume and ICH-induced motor impairment in the contralateral-to-ICH forelimb, even in the chronic assessment period [35].

### Horizontal ladder task

Animals were trained on the horizontal ladder task in order to assess ICH-induced motor impairment through another modality [36]. The horizontal ladder task was selected as a secondary endpoint because we have previously shown that this task is reasonably sensitive to the relationship between lesion volume and ICH-induced motor impairment (though, less sensitive than the staircase task [35]). Animals were given the opportunity to cross the apparatus 5 times a day on the two days prior to surgery. Testing sessions involved videotaping the animals crossing a 0.5 m section of the ladder over 2 to 4 consecutive trials. Videos were analyzed using previously published methods [35]. Our analysis focused on the contralateral-to-ICH forelimb. Baseline testing occurred prior to ICH surgery. The percent of successful steps was calculated for the sum of 2 to 4 trials (number conducted per test day) according to the following formula, and averaged across trials:

$$\%\;Successful\;steps=\frac{\#of\;successful\;steps}{\#of\;total\;steps}\times 100$$

#### Study 2A (R18D)

Post-ICH horizontal ladder testing occurred on day 7, 14, 21 and 28. Ladder rungs were variably spaced 1–3 cm.

#### Study 2B (R18)

Post-ICH horizontal ladder testing occurred on days 7 and 21 only. Ladder rungs were variably spaced 1–5 cm. Task difficulty was increased in Study 2B by increasing the spacing between ladder rungs because of the lack of functional impairment observed in Study 2A.

### Histology

On day 28 following ICH, animals were overdosed using a 100 mg/kg intraperitoneal dose of sodium pentobarbital. The animals were then transcardially perfused with 0.9% saline and 10% neutral buffered formalin. We obtained 40 μm thick coronal brain sections every 200 μm through the brain using a cryostat. Sections were stained with cresyl violet and lesion volume was analyzed using ImageJ software (version 1.51; NIH) according to our published protocol [35].

### Statistical analysis

All data were analyzed using R statistical software (Version 3.5.1; Vienna, Austria). Lesion volume and hematoma volume were analyzed using one-way ANOVA, with planned Tukey post-hoc comparisons where appropriate. Behavioural endpoints were analyzed using two-way (mixed) ANOVA. Assumptions of ANOVA were checked in all analyses, and appropriate non-parametric statistics (e.g., Kruskal-Wallis) or corrections (e.g., Greenhouse-Geisser) were used when assumptions were violated. All data were considered to be statistically significant at p < 0.05. All data are presented as mean ± 95% confidence interval (CI).

In order to fully address the safety and efficacy of R18 and R18D, we conducted a series of post-hoc pooled analyses on the hematoma volume and lesion volume data across experiments.

## Results

### Exclusions

#### Study 1 (hematoma assessment)

In Study 1A, one rat was excluded from the saline group due to surgical error. In Study 1B three rats were excluded, one each from the R18 300 nmol/kg, R18 1000 nmol/kg, and R18D 300 nmol/kg groups; one was due to surgical error, and two were due to technical error during tissue processing.

#### Study 2 (histology/functional assessment)

There was only one behavioural exclusion in Study 2: during horizontal ladder assessment in Study 2A, data could not be obtained from one animal in the 300 nmol/kg group due to failure to cross the apparatus. During the histological assessment in Study 2A, one rat was excluded in the saline group, and three rats in the R18D 300 nmol/kg group were excluded due to technical error during tissue processing. In Study 2B, one animal in the saline group was excluded from lesion volume analysis due to technical error during tissue processing. As decided *a-priori*, we analyzed the remaining data in cases where some data had to be excluded (e.g., behavioural data were included despite loss of histology).

### Results

#### Study 1: Hematoma assessment

**Fig 2:**
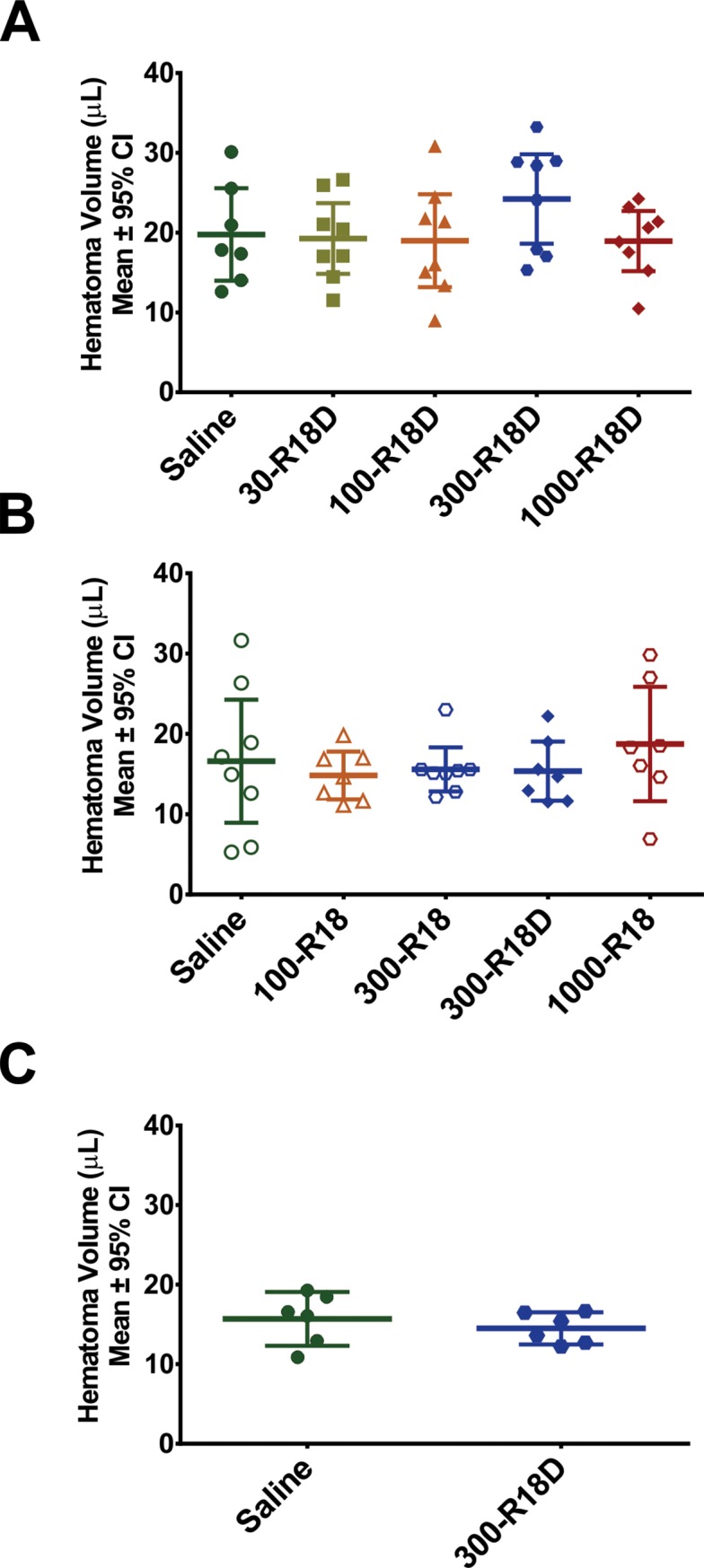
Results from biochemical hematoma volume assessment at 24 hours post-ICH. Hematoma volume (μL) data for Studies 1A-C. Data showed that bleeding was not significantly impacted for any dose of R18D (A and C: batch 1 of peptide; B: batch 2) or R18 (B) when administered 30-minutes post-ICH. Filled-shapes denote doses used in R18D studies, open-shapes denote doses used in R18 studies.

R18D (batch 1 and batch 2) and R18 did not have a statistically significant impact on bleeding when administered 30 minutes post-collagenase infusion and following a 24-hour survival period (Fig 2A–2C).

#### Study 2: Lesion volume assessment

**Fig 3:**
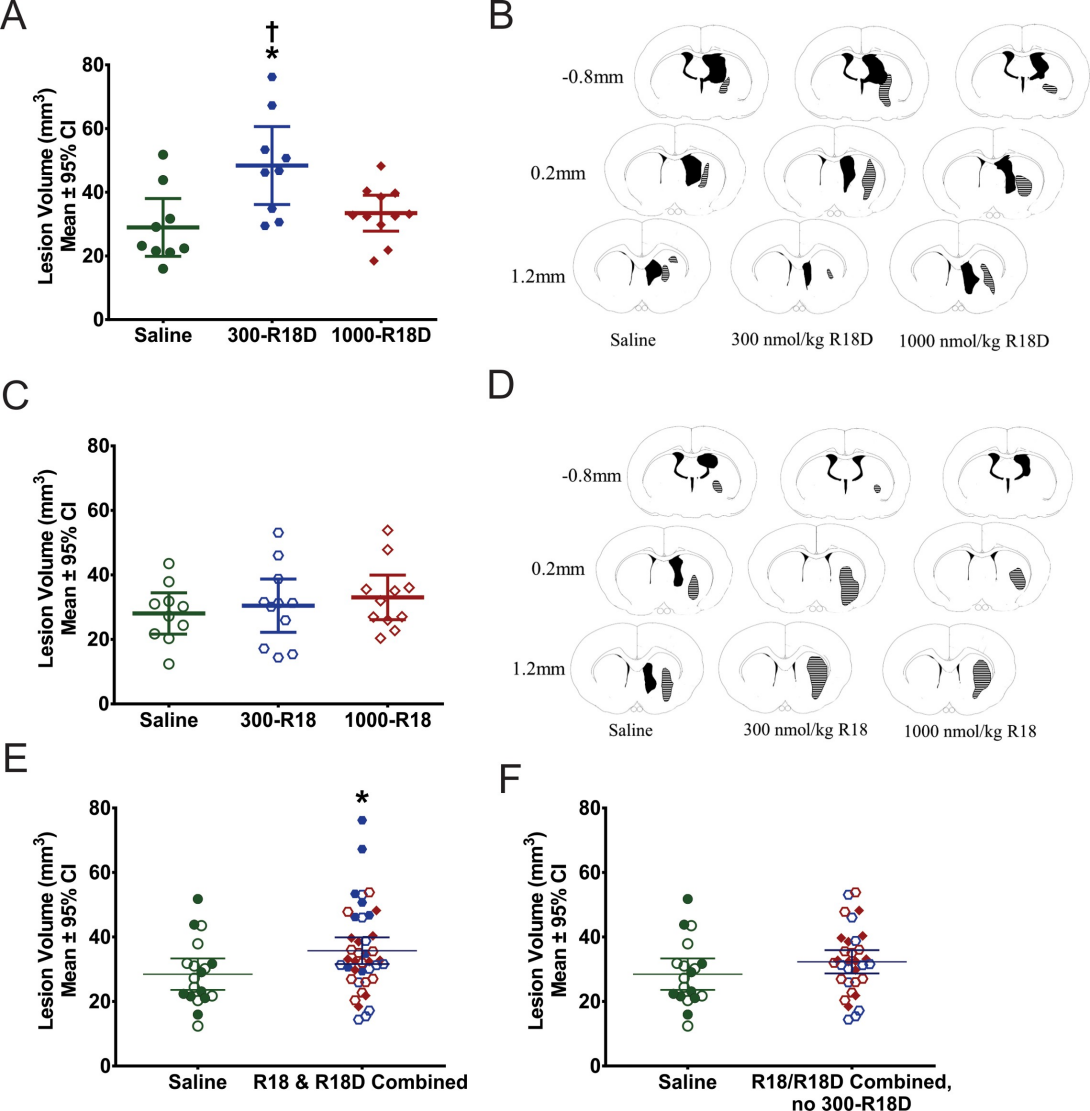
Tissue loss at 28 days post-ICH was largely confined to the basal ganglia. **A.** In Study 2A animals in the 300 nmol/kg R18D group had significantly more tissue loss than saline control animals (denoted by the asterisk), and 1000 nmol/kg R18D group (denoted by the cross). Rats in the 1000 nmol/kg group did not differ from saline controls. **B.** Representative histological images from 28 days post-ICH are shown for rats in each treatment group in Study 2A. The black areas represent ventricles, and the shaded areas represent the lesion. **C.** In Study 2B there were no significant differences in tissue loss between the groups. **D.** Representative histological images from 28 days post-ICH are shown for animals in each treatment group in Study 2B. The black areas represent ventricles, and the shaded areas represent the lesion. **E.** Post-hoc pooled analysis of all treatment conditions from Study 2A and Study 2B are shown with respect to lesion volume. When all doses of R18 and R18D were combined across studies and compared to all controls across studies, there was significantly more tissue loss in treated animals (denoted by the asterisk). **F**. All treatment doses combined between Study 2A and 2B are shown except 300 nmol/kg R18D. The significant effect observed in panel E does not persist when the 300 nmol/kg R18D group is removed from the analysis. **A, C, E, F:** Filled-shapes denote doses used in R18D study, open-shapes denote doses used in R18 study.

R18D (batch 1) at the 300 nmol/kg dose, but not at the 1000 nmol/kg dose, statistically increased lesion volume compared to the saline control (Fig 3A; p = 0.0056) when assessed at 28 days post-ICH.

R18 at the 300 and 1000 nmol/kg dose did not statistically alter lesion volume compared to the saline controls when assessed at 28 days post-ICH (Fig 3C). Representative histological images can be found in S1 Fig.

#### Study 2: Functional assessment

**Fig 4:**
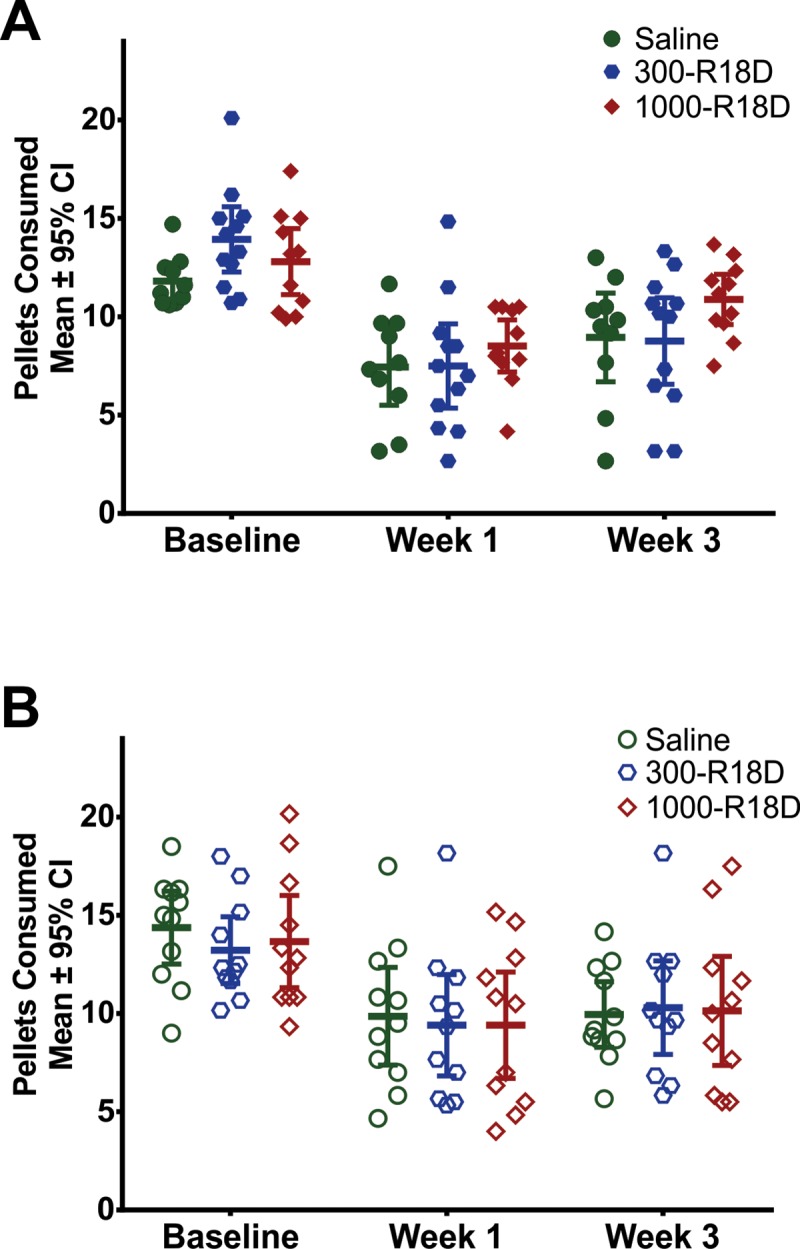
Results of the Montoya staircase forelimb assessment are shown for Study 2A with R18D (**A**) and 2B with R18 (**B**). In both studies, there was statistically significant post-ICH skilled-reaching impairment that persisted for 3 weeks (Cohen’s d = 1.25 in R18D study, 1.16 in R18 study), but no statistically significant difference between groups. There were no baseline differences between groups in either experiment (Study 2A: p = 0.10; Study 2B: p = 0.65). Post-hoc analyses revealed significantly lower skilled reaching performance for each pairwise comparison to baseline (p < 0.05 for all groups at all time points). Filled-shapes denote doses used in the R18D study, open-shapes denote doses used in the R18 study.

Staircase test: The staircase forelimb reaching test revealed a statistically significant effect of time in both Study 2A and Study 2B (Fig 4A and 4B; p < 0.001), indicating ICH-induced skilled-reaching impairment in the contralateral-to-ICH forelimb. In addition, post-hoc tests in both studies confirmed that there were statistically lower reaching values for all groups at week 1 and 3 post-ICH (p < 0.05 for all pairwise comparisons to baseline), indicating persistent skilled-reaching deficits.

In sum, a single dose of R18D (300 or 1000 nmol/kg) or multiple doses of R18 (300 or 1000 nmol/kg) commencing 30-minutes post-collagenase injection did not statistically change forelimb reaching (p = 0.24 and p = 0.94, respectively) compared to saline treatment; the interaction was not statistically significant in either part of Study 2 (Study 2A, p = 0.22; Study 2B, p = 0.85).

Horizontal ladder test: In Study 2A (R18D), the horizontal ladder test revealed no main effect of time (p = 0.26; S2A Fig) or group (p = 0.95). The interaction was statistically significant (p = 0.045), but time comparisons to baseline showed that no deficits were evident (p ≥ 0.093). This means that the horizontal ladder task was unable to detect stroke-induced motor impairment, and therefore unable to detect any potential treatment effects.

In Study 2B (R18) the horizontal ladder test did not reveal a statistically significant effect of time, despite increasing the task difficulty compared to Study 2A (p = 0.18; S2B Fig). The effect of group was not statistically significant (p = 0.21). The interaction was not statistically significant (p = 0.08).

## Post-hoc pooled analyses

### Exploratory pooled hematoma volume analyses

#### All R18D doses pooled

When all doses (and batches) of R18D (Study 1 A, B and C) were pooled, and hematoma volumes were compared to saline controls (from all studies), there was no statistically significant effect of R18D on hematoma volume (p = 0.60).

#### All R18 doses pooled

When all doses of R18 (from Study 1B) were pooled and hematoma volumes were compared to saline controls (from all studies), there was no statistically significant effect of R18 on hematoma volume (p = 0.27).

#### R18D and R18 combined

When all treatments of R18 and R18D were pooled across all studies, and hematoma volumes were compared to saline controls from all studies, there was no statistically significant effect of the combined treatments on hematoma volume (p = 0.69). Additionally, post-hoc region of practical equivalence (ROPE) analysis and bootstrapped effect size analyses yielded the same conclusions (data not shown).

### Exploratory pooled lesion volume analyses

#### All R18D doses pooled

When all doses of R18D were pooled from the efficacy phase of Study 2A and lesion volume was compared to saline controls from Study 2A, there was statistically more tissue loss in the treated animals (p = 0.049; Cohen’s d = 0.86, large effect size).

#### R18D and R18 combined

When tissue loss for all doses of R18D (from Study 2A) and R18 (from Study 2B) were compared to saline controls for both studies, there was statistically more tissue loss in treated animals compared to controls (p = 0.037; Cohen’s d = 0.62, medium effect size; Fig 3 Panel E). Additionally, post-hoc ROPE analysis and bootstrapped effect size analyses yielded the same conclusions (data not shown). When the 300 nmol/kg R18D group was removed from the analysis, the effect was eliminated (p = 0.20; Fig 3F).

## Discussion

CARPs, which include short chain polymers of arginine, have shown evidence of neuroprotective efficacy in *in vitro* [39] and *in vivo* excitotoxicity [40] models and in several models of ischemic [7–9] and hemorrhagic stroke (Table 1). CARPs have also demonstrated neuroprotective efficacy in models of traumatic brain injury [12]. CARPs produce neuroprotection by acting on several potential mechanisms [13–16]. Overall, the data collected in this study suggest that, at the doses examined, poly-arginine peptides R18 and R18D were mostly safe in that they did not exacerbate bleeding, but do not produce histological or behavioural benefits when administered at various dosing schedules following collagenase-induced ICH in rats. The latter findings were surprising given that other CARPs have demonstrated the capacity to improve functional outcomes and reduce neuronal injury in rodent ICH models (Table 1). There are several reasons that could account for the contrasting results obtained in this and other studies including differences in ICH models, peptide dose and dosing regimens, histological assessments (e.g. Nissl staining vs TUNEL staining) and functional assessments (e.g. staircase test vs water maze) used, and peptide efficacy at targeting post-ICH injury processes [41,42]. Finally, we did not deem a sham control group to be necessary. Because we collected longitudinal data, we were able to assess the behavioural deficits experienced by the animals as a change from baseline functioning. In general, sham-operated animals experience minimal brain damage and slight weight-loss which typically resolves over 24 hours, and little to no behavioural deficits [35].

Notably, none of the R18 and R18D treatment doses used in this study impacted ongoing bleeding when assessed using a biochemical hemoglobin assay [32], despite having sufficient (82%) observed statistical power to detect a 25% (~5 μL) change in hematoma volume. It is possible that smaller changes occurred, but our study was not sufficiently powered to detect group differences that small. This result has high future clinical relevance, as it indicates that R18 peptides may be safe to administer before stroke subtype has been determined, opening up the possibility of early field administration by ambulance paramedic or at a remote hospital before patient transport to a tertiary hospital. An early neuroprotective intervention has the advantage of maximising the preservation potential for salvageable tissue (i.e. slow infarct growth) in the ischemic penumbra in patients suitable for thrombolysis and/or thrombectomy, as well as patients ineligible for these recanalization therapies. Of note, the 30 nmol/kg dose of R18 was not included in Study 1B due to the fact that 30 nmol/kg R18D did not have a significant impact on hematoma volume in Study 1A and due to the inferior *in vivo* stability of L-peptides compared to D-peptides. Because R18 has less in-vivo stability than R18D, we opted to select higher concentration doses for Study 1B; for the same reason, we opted to make use of repeated doses per animal in Study 2B as opposed to a single dose per animal as in Study 2A.

As mentioned above, hematoma volume was selected as an endpoint because it is a robust determinant of mortality and morbidity in ICH patient populations [6]. Our assessment of safety based on hemorrhage volume (i.e., potential bleeding complications) provide strong evidence that R18 and R18D do not worsen post-ICH outcomes by inducing coagulopathy when administered during a period of ongoing bleeding. In addition, the collagenase model of ICH is an ideal model to investigate whether ongoing bleeding can be aggravated by a putative treatment because the timing of ictus is controlled, and the profile of bleeding is similar to the profile of bleeding in patients, which in some patients, evolves over hours [43–45].

Relatedly, hemorrhage occurs as a result of hemorrhagic cerebral infarction and as a bleeding complication following reperfusion therapy in ischemic stroke (i.e., hemorrhagic transformation) with devastating consequences [46]. While hemorrhagic infarction typically occurs 1–2 weeks after ischemic stroke, in about 9% of patients it occurs in the first 24 hours [47]. Hemorrhagic transformation is not uncommon in the acute period of ischemic stroke, occurring in up to 40% of clinical cases and in up to 71% of autopsy cases [48,49]. The incidence of any hemorrhagic transformation can increase to 60% when endovascular therapies are used to remove or dissolve the clot, depending on the mechanical thrombectomy device and route of tissue plasminogen activator (tPA) administration [50]. Symptomatic hemorrhagic transformation occurs in about 6–15% of patients receiving endovascular therapy, compared to 0.6% receiving clinical management alone [51]. Symptomatic hemorrhagic transformation can lead to significant neurological deterioration and is strongly associated with mortality [52]. Because R18 and R18D have shown neuroprotective efficacy in models of ischemic stroke, our data suggest that a subset of individuals with hemorrhagic complications following cerebral ischemia may not have worsened conditions as a side effect of treatment with these agents. In summary, any agent with the potential to significantly impact bleeding should be thoroughly assessed and complications must be understood or ruled out.

An interesting finding of this study was the significantly higher lesion volume in animals treated with R18D at the 300 nmol/kg dose, despite animals treated with the higher 1000 nmol/kg dose demonstrating a lesion volume similar to saline treated animals (Study 2A). We cannot ascertain whether this was a true effect or false positive without additional study. Therefore, caution must be taken in interpreting our results, as variability is known to be relatively high in preclinical ICH research [45]. Indeed, given that the higher (1000 nmol/kg) dose of R18D and other studies using CARPs at high doses do not appear to increase lesion volume, it seems the latter possibility is more likely (i.e., Type I error). In addition, we used R18D at the 300 nmol/kg dose in Studies 1 A and C to explicitly rule out the possibility of coagulopathy contributing to worsened histological tissue loss that was observed in Study 2A, due to the observation of a slightly larger hematoma volume in the 300 nmol/kg R18D group in Study 1A. The results of Study 1 B and C confirm that this was likely to be a spurious observation. Finally, lesion volume differences were not observed with R18 (Study 2B) despite the peptide being administered multiple times over 7 days, as opposed to a single dose for R18D. We believe, from a perspective of safety testing, that repeated dosing over multiple days would more likely result in an adverse effect if there was one. However, there is a possibility that this difference could be due to the chemical properties of R18D increasing its *in vivo* enzymatic stability [53]. Ultimately, additional replicative studies are needed to make any conclusive statements about the impact of R18D administered in the acute period of ICH on long-term histological outcomes.

We did not detect improved (or worsened) behavioural outcomes, as assessed by the Montoya staircase task [34]. We observed significant impairment in post-ICH reaching on this task for 3 weeks, which was not improved by the R18D and R18 peptide treatments administered in this study. We do not believe that this is due to a ceiling effect in this task post-ICH as each treatment group mean at the 1- and 3-week time-points were well below the 21-pellet maximum that could be obtained on this test.

We used the horizontal ladder task as another modality to assess forelimb motor impairment, and were unable to detect any ICH-related impairment, despite using similar protocols to those that we have used in the past [35]. We expect that a modest lesion volume is the most likely explanation for our inability to detect ICH-related impairment on the horizontal ladder task, and we have previously shown that this task is only moderately sensitive to striatal lesion volume (e.g., Spearman’s rho ~ 0.40). We did not have the same range of lesions in the current study as we did in our previous publication, and observed correlations were smaller (Pearson’s r = 0.28) between lesion volume and horizontal ladder walking performance.

We chose not to use similar behavioural tasks to those that have been used in previous literature (such as those in Table 1) because our study was designed to understand whether R18 and R18D conferred chronic histological and functional benefits for weeks following ICH. Indeed, we and others have shown that tests such as the rotarod and Morris water maze can detect *whether* an ICH has occurred, but are unable to differentiate between large and small lesions (i.e., the difference between an untreated and a treated ICH) [35,54]. On the other hand, we have shown that the Montoya staircase task (and to a lesser extent, the horizontal ladder task) can reliably discern between large and small lesion volumes at the group level in the chronic post-ICH period [35]. Although it is certainly possible that additional tests (e.g., rotarod, water maze) may have detected treatment effects, we used tests that we have extensive experience with, and have proven effectiveness in picking up neuroprotective effects [35,43,55]. Finally, we note that the peptide dosing regimens are not directly comparable between Study 2A and 2B. We therefore acknowledge that conclusions with respect to our data must be understood within the context of each individual study, in particular with respect to the dosing regimen used.

## Limitations and future directions

As mentioned, previous studies using other CARPs, including the TAT-NR2B9c peptide, have shown efficacy in ICH models, and therefore it would have been useful to compare the effects of R18/R18D with TAT-NR2B9c, which is in clinical trials for acute ischemic stroke, or the CN-105 peptide, which is being developed as a potential treatment for ICH and has recently passed a Phase 1 clinical trial [11,56]. Future studies should also make use of additional ICH models with longer-term behavioural and more comprehensive histological assessments, as well as a wider range of ICH sizes to clarify some of the findings discussed herein. In addition, the use of MRI may be useful in monitoring the changes in the peri-hematoma zone and detecting treatment effects of peptides. Finally, future studies could investigate whether CARPs interact with thrombolytic therapies and have any effects on the risk of hemorrhagic transformation after cerebral infarction.

## Conclusion

When given in the acute period of collagenase-induced ICH, neither R18 nor R18D exacerbated bleeding, which suggests that the peptides are likely to be safe when administered to patients before stroke subtype has been determined and in patients with hemorrhagic stroke. However, additional studies are required in the present and other ICH models and rigorous assessment of different dosage strategies and outcome protocols are necessary to confirm the safety of the peptides and to determine the potential behavioural or histological efficacy of CARPs on outcomes in ICH.

## Supporting information

S1 Fig Representative histological images from an R18 rat (1000 nmol/kg group). This animal had a 35.6 mm^3^ loss of tissue. The border between damaged tissue (now a cavity) and normal brain is readily apparent. Images were taken at **A)** 0.8 mm, **B)** -0.2 mm, and **C)** -1.2 mm to Bregma using a Leica DB6B microscope with 5× (**left)** and 20× (right) objectives and a DFC7000T camera. Images were stitched together in Leica Application Suite X (LAS X). Scale bars for left images represent 1 mm and 0.25 mm for images on the right. Similar MRI, light microscopic, and ultrastructural images can be found in previously published work by our laboratory and others [27,37,38]. (S1 Fig).
(TIF)

S2 Fig Horizontal ladder data are shown for Study 2A with R18D (**A**) and Study 2B with R18 (**B**). Study 2A used ladder rung spacing from 1–3 cm and testing was performed at weeks 1, 2, 3, 4; Study 2B used ladder rung spacing from 1–5 cm and testing was performed only at weeks 1 and 3. This task was not sensitive to ICH-induced motor impairment in either study. Filled-shapes denote doses used in the R18D study, open-shapes denote doses used in the R18 study. BL = baseline; W1, W2, W3, W4 = week 1, week 2, week 3, week 4, respectively. (S2 Fig)
(TIF)

S1 Data Data for all experiments have been made available as supplementary material. (S1 Dataset).
(XLSX)
