# Supplementary figures and images for: Poly-arginine-18 peptides do not exacerbate bleeding, or improve functional outcomes following collagenase-induced intracerebral hemorrhage in the rat
Source: PLoS One. 2019 Nov 7;14(11):e0224870. doi: 10.1371/journal.pone.0224870 (PMC6837498; doi:10.1371/journal.pone.0224870)

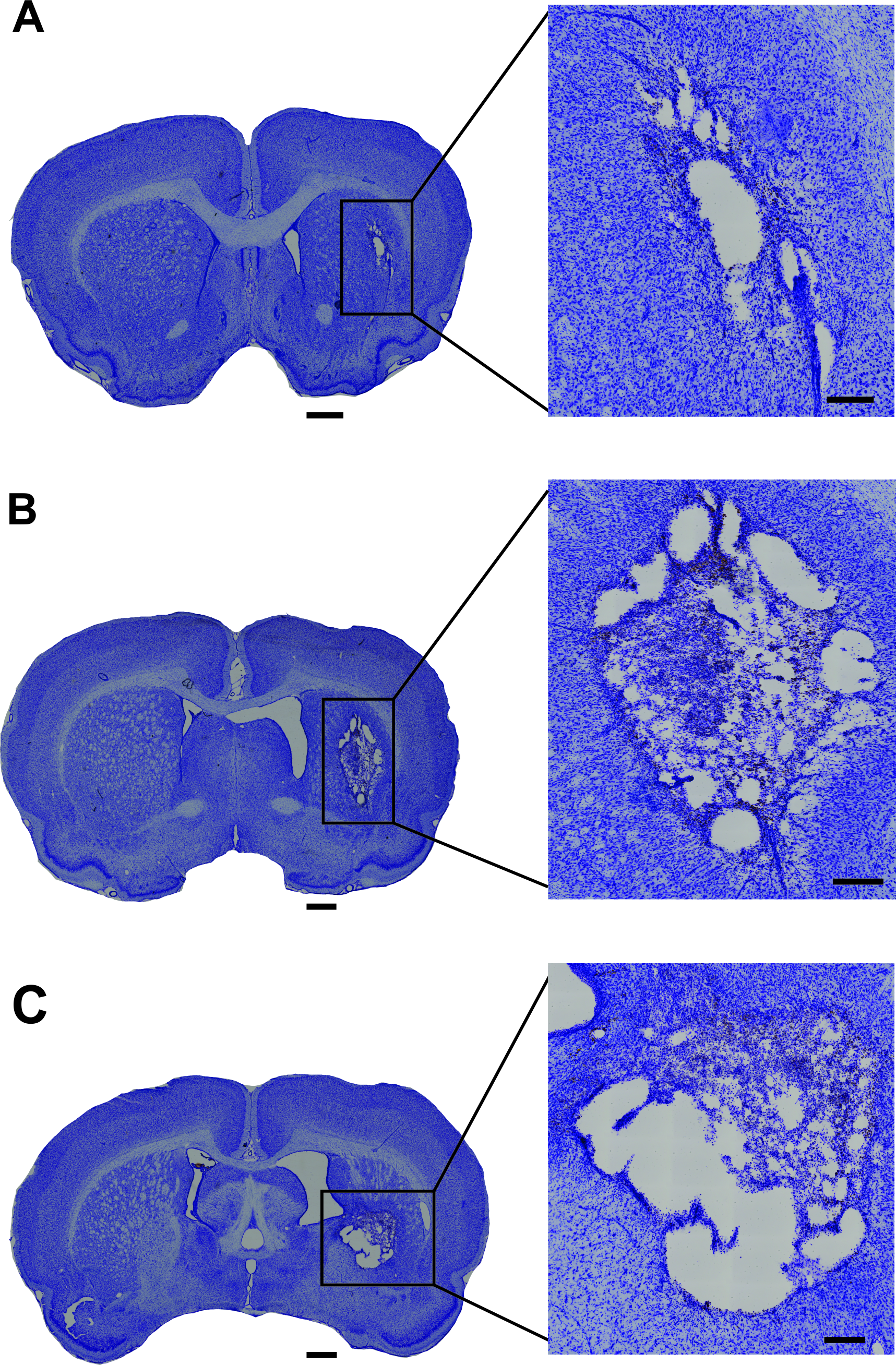

Supplement: S1 Fig — This animal had a 35.6 mm3 loss of tissue. The border between damaged tissue (now a cavity) and normal brain is readily apparent. Images were taken at A) 0.8 mm, B) -0.2 mm, and C) -1.2 mm to Bregma using a Leica DB6B microscope with 5× (left) and 20× (right) objectives and a DFC7000T camera. Images were stitched together in Leica Application Suite X (LAS X). Scale bars for left images represent 1 mm and 0.25 mm for images on the right. Similar MRI, light microscopic, and ultrastructural images can be found in previously published work by our laboratory and others [27,37,38]. (S1 Fig). (TIF) [file pone.0224870.s001.tif]
